# Multifactor transcriptional profiling of potato during 2,4-D-induced resistance to common scab disease

**DOI:** 10.3389/fpls.2025.1641317

**Published:** 2025-08-18

**Authors:** Matthew L. Fabian, Hien P. Nguyen, John R. Stommel, Christopher R. Clarke

**Affiliations:** Genetic Improvement for Fruits and Vegetables Laboratory, Beltsville Agricultural Research Center, U.S. Department of Agriculture-Agricultural Research Service, Beltsville, MD, United States

**Keywords:** potato common scab, *Streptomyces*, 2,4-D, transcriptional response, disease management

## Abstract

Foliar application of low-dose 2,4-dichlorophenoxyacetic acid (2,4-D) has been demonstrated to reduce potato common scab disease caused by phytopathogenic *Streptomyces*. Foliar-applied 2,4-D is translocated to the tubers but does not cause direct toxicity against the pathogen. The efficacy of 2,4-D treatment for common scab disease management is inconsistent among field trials in the literature, and the exact mode of action is unknown. Here, we identified transcriptomic responses of potato to low-dose 2,4-D treatment in the presence and absence of the pathogen and in tuber periderm and foliar tissue. Pathogen infection primarily altered transcriptomic responses in tuber periderm tissue, while foliar 2,4-D application caused larger shifts in gene expression in leaf tissue, as expected. Gene ontology (GO) terms associated with pathogen defense, stress responses, and enzymatic inhibitors were significantly enriched among differentially expressed genes in the tuber response to the pathogen. There were more differentially expressed genes and enriched GO terms in response to the pathogen when plants were treated with 2,4-D than in the non-2,4-D-treated plants, including differentially expressed genes and GO terms related to lipases, jasmonic acid signaling, and transport. Fewer differentially expressed genes were identified in tuber tissue than in leaf tissue following foliar 2,4-D treatment, but GO terms related to sucrose transport were enriched in tuber RNA samples from 2,4-D-treated, non-inoculated plants. Altered glucose and fructose, but not sucrose, levels in tuber medulla and periderm tissue, the site of common scab infection, were observed in 2,4-D-treated plants. Utilizing multiple factors, i.e., mock or 2,4-D treatments in both the presence and absence of the pathogen, in parallel transcriptional profiling experiments enabled the identification of pathways that directly respond to 2,4-D treatment in both foliar and tuber tissue and pathways with altered response in the context of pathogen infection. Identifying tools to more consistently induce these changes may enable more robust disease management than indirect foliar 2,4-D treatments.

## 1 Introduction

Common scab of potato is an economically costly disease of potato (*Solanum tuberosum*) caused by more than 10 species of *Streptomyces* that produce the phytotoxin thaxtomin A (Francis et al., 2010; Li et al., 2019; Thapa et al., 2019). While other virulence determinants are present in *Streptomyces* common scab pathogens, thaxtomin A and related toxins are the primary compounds responsible for the manifestation of the raised and pitted lesions associated with common scab of potato and other root and tuber crops (Clarke et al., 2022; Weisberg et al., 2023; Zhang et al., 2024), and the abundance of thaxtomin A-producing bacteria has been shown to be directly correlated with common scab disease severity (Shelley et al., 2023). Accordingly, much research has focused on the regulation and biosynthesis of thaxtomins by *Streptomyces* bacteria (Loria et al., 2008; Li et al., 2019), and comparatively little is known about the passive and active defenses responsible for host resistance to common scab. RNA sequencing has recently been used to identify transcriptomic responses to common scab infection in potato and has facilitated the identification of genes differentially regulated between susceptible and resistant cultivars (Fofana et al., 2020; Li et al., 2024), including genes putatively involved in tryptophan-induced common scab resistance (Zhao et al., 2022).

Disease management options for common scab are limited and inconsistent in their efficacy (Dees and Wanner, 2012; Braun et al., 2017). Cultural management strategies, including altered soil pH (Waterer, 2002; Marques et al., 2021), high soil moisture (Lapwood et al., 1973; Davis et al., 1976; Wilson et al., 2001; Johansen et al., 2015), and crop rotation (Larkin et al., 2011; Hunjan and Sabhikhi, 2020), have been tested, yielding inconsistent results and limited agronomic practicality. Chemical treatments such as pentachloronitrobenzene, as well as fumigants, including chloropicrin, have demonstrable efficacy for common scab disease management (Menzies, 1957; Emden and Labruyère, 1958; Rosser, 1960; Davis et al., 1976; Wilson et al., 1999; Al-Mughrabi et al., 2016) but are considered to also have substantial negative externalities, including detrimental impacts to soil health. Furthermore, several fungicides such as mancozeb and fluazinam have been reported to decrease common scab disease severity; however, field trial results are inconsistent (Mcintosh, 1973; McIntosh, 1977; Wilson et al., 1999; Al-Mughrabi et al., 2016).

Surprisingly, low-dose chemical treatments with the synthetic auxin herbicide 2,4-dichlorophenoxyacetic acid (2,4-D) and the related compound 3,5-D have been reported to attenuate disease severity for multiple cultivars of potato but also with inconsistent efficacy across trials (McIntosh et al., 1981, 1982, 1985; Waterer, 2010; Thompson et al., 2014b; Clarke et al., 2020). Treatment with 2,4-D has also been shown to enhance resistance to additional pathogenic diseases in tuber, including powdery scab (Thompson et al., 2014a; Clarke et al., 2020). Efficacy appears to be dependent on applying the foliar treatment at or shortly before tuber initiation (Thompson et al., 2013). Foliar 2,4-D treatments have been characterized for various physiological impacts on potato, including enhanced anthocyanin content in red potato skin (Fults et al., 1950; Busse and Bethke, 2020), as well as changes in sugar content (Payne and Fults, 1955). Foliar 2,4-D treatments at physiologically relevant doses associated with these phenotypic shifts have been associated with no impact, a small positive impact, or a small negative impact on total potato yield (McCubbin, 1957; Wort, 1965; Busse and Bethke, 2020; Clarke et al., 2020; Qin et al., 2021).

The mechanisms through which foliar treatment of 2,4-D leads to inhibition of common scab disease progression in potato tubers remain uncertain. Following foliar applications, 2,4-D and related compounds have been observed to translocate to and accumulate in tubers (Burrell, 1982; Tegg et al., 2008). Accumulation of 2,4-D in tubers has been associated with reduced phytotoxicity of thaxtomin A (Tegg et al., 2008, 2012), but physiologically relevant doses of 2,4-D have been shown to not directly interfere with thaxtomin A-induced tuber necrosis (Clarke et al., 2020). Furthermore, foliar 2,4-D treatments do not lead to notable changes in periderm or lenticel development, which are considered critical physiological features mediating *Streptomyces* infection of tubers (Tegg et al., 2008). While 2,4-D has been shown to be directly inhibitory to pathogen growth and expression of pathogenicity factors in other pathosystems (Duan et al., 2023), physiologically relevant concentrations of 2,4-D inhibit neither *Streptomyces* growth nor biosynthesis of thaxtomin A (McIntosh et al., 1981; Tegg et al., 2008). Therefore, 2,4-D likely suppresses common scab through an indirect mechanism. One proposed mechanism is 2,4-D-induced inhibition of thaxtomin A uptake and transport in plant cells through interference with auxin transporters. In support of this hypothesis, multiple genetic studies have demonstrated that plant genotypes with hypersensitivity to thaxtomin A are also hypersensitive in their response to auxin transport inhibitors such as 1-naphthylphthalamic acid (Tegg et al., 2013, 2016).

There are several other examples of low-dose herbicide treatments that induce disease resistance, often through unknown mechanisms (Grinstein et al., 1984; Awadalla and El-Refai, 1992; Ueno et al., 2004; Martinez et al., 2018). Because insights into the mechanisms underlying 2,4-D-induced resistance to common scab in potato are limited, in this study, we utilized a multifactor experimental approach to classify the transcriptional responses, in both potato leaf and tuber tissue, to low-dose foliar sprays of 2,4-D in the context of common scab infection.

## 2 Materials and methods

### 2.1 Plant material, *Streptomyces* inoculation, and 2,4-D treatment for pathogenicity assays and RNA sequencing

Twenty-four cultivar RH89-039-016 (RH89) potato plants (The Potato Genome Sequencing Consortium, 2011; tissue culture plants originally obtained from Wageningen University) were grown in 6-in. greenhouse pots, and one-half were inoculated with *Streptomyces caniscabiei* sp. nov. NE06-02D (“*Strep*”) at 2 × 10^6^ CFU/pot as previously described (Weisberg et al., 2021) for the RNA sequencing experiment. At 4 weeks after planting (wap), six inoculated and six non-inoculated plants were sprayed until runoff with 200 mg L^−1^ of Weedar 64 2,4-D (Nufarm, Alsip, IL, USA) supplemented with 0.05% Tween 80 (Sigma-Aldrich, St. Louis, MO, USA). The remaining 12 plants (six inoculated and six non-inoculated) were sprayed with a mock treatment of water supplemented with 0.05% Tween 80. At 12 wap (8 weeks after 2,4-D treatments), tubers from all treatment groups were harvested for the collection of tuber periderm tissue for RNA extraction and scoring of disease severity as previously described (Clarke et al., 2020).

### 2.2 RNA extraction, sequencing, and analysis

For each of the four treatment conditions (“mock,” non-inoculated and mock-treated; “2,4-D,” non-inoculated and 2,4-D-treated; “*Strep*,” inoculated and mock-treated; “2,4-D + *Strep*,” inoculated and 2,4-D-treated), three biological replicates of tuber periderm tissue and three biological replicates of leaf tissue from a single experiment were sampled (except as noted in **Supplementary Table S1**). Each biological replicate consisted of samples pooled from two plants, with six total plants used for each treatment group. Samples were stored at −80°C. Leaf samples were taken 2 days post-2,4-D treatment, and tuber samples were taken at harvest (8 weeks post-treatment). For each sample, 100 mg of tissue was ground in liquid nitrogen using a mortar and pestle, then vortexed in 1 mL of TRI Reagent (Sigma-Aldrich). RNA extraction was conducted via RNEasy Mini Kit with on-column RNase-Free DNase Set (Qiagen, Hilden, DE) with modifications. After incubation for 30 min at room temperature (RT), samples were vortexed in 600 µL of Buffer RLT supplemented with 6 µL of β-mercaptoethanol, then incubated at RT for 5 min. Samples were shaken for 1 min with 200 µL of chloroform, then incubated at RT for 10 min, followed by centrifugation at 14,000 rpm for 30 min at 4°C; this process was repeated using 1 mL of the resulting aqueous phase. Six hundred μL of aqueous phase was gently mixed with 300 µL of 100% ethanol and transferred to RNeasy Mini spin columns, with the remaining steps carried out per manufacturer’s instructions.

The RNA sequencing pipeline was conducted by Azenta Life Sciences (South Plainfield, NJ, USA). RNA samples were quantified via Qubit 2.0 Fluorometer (Thermo Fisher Scientific, Waltham, MA, USA), and RNA integrity was verified via 4200 TapeStation (Agilent Technologies, Palo Alto, CA, USA). RNA sequencing libraries were prepared with the NEBNext Ultra II RNA Library Prep Kit for Illumina using the manufacturer’s instructions (New England Biolabs, Ipswich, MA, USA). Sequencing libraries were pooled and clustered on two lanes of a flow cell and loaded onto the Illumina HiSeq 4000 or equivalent (Illumina, San Diego, CA, USA). Samples were sequenced using a 2 × 150-bp paired-end (PE) configuration. Image analysis and base calling were conducted using HiSeq Control Software. Raw sequence data generated from Illumina HiSeq were converted into FASTQ files and de-multiplexed using Illumina’s bcl2fastq 2.17 software. One mismatch was allowed for index sequence identification.

From the resulting paired-end FASTQ files, Illumina adapter and leading 15-bp sequences were trimmed from reads via Trim Galore (https://www.bioinformatics.babraham.ac.uk/projects/trim_galore/) with default parameters and verified via FastQC reports for analysis of FASTQ file quality. For alignment of trimmed reads, genome indexing and mapping to the reference genome was performed via HISAT2 (https://daehwankimlab.github.io/hisat2/) with default options and utilized the DM 1-3–516 R44 (NCBI accession number PRJNA63145) annotated genome v6.1 (https://spuddb.uga.edu/dm_v6_1_download.shtml). Output SAM files were converted to BAM format, and mapping quality was analyzed via Samtools (http://www.htslib.org/). The High Confidence Gene Model Set annotations file for DM v6.1, in GFF3 format, was converted to GTF format via gffread (http://ccb.jhu.edu/software/stringtie/gff.shtml). For the alignment of reads to gene models, featureCounts (http://subread.sourceforge.net/) was employed using the representative, high-confidence gene model GTF and with the following options: -p (paired-end FASTQ files), –countReadpairs (count read pairs), and –transcript (assign reads at transcript-level). For pseudoalignment of trimmed reads, Salmon (https://combine-lab.github.io/salmon/) was employed to index, and assign read pairs to, the representative, high-confidence cDNA sequences for DM v.6.1.

**Figure 1:**
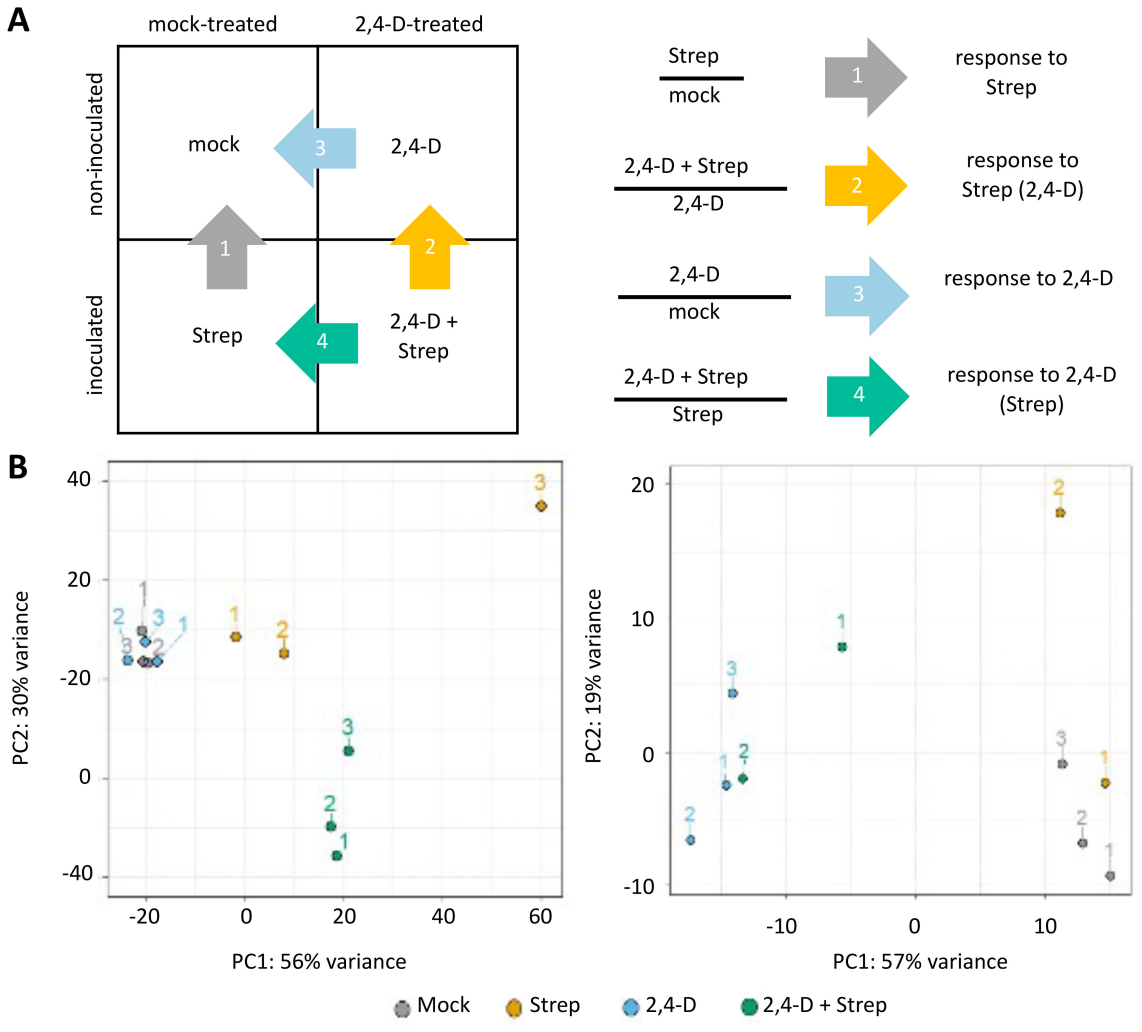
RNA-seq quality control and schema. **(A)** Schema for the comparison groups for RNA-seq differentially expressed gene (DEG) analysis. **(B)** Principal component analysis (PCA) of RNA-seq read pairs, assigned to genome features, by tuber (left) and leaf (right) samples.

DEG modeling and estimate dispersion analysis and principal component analysis (PCA) of assigned read pairs were performed via the “DESeq2” package (Love et al., 2014) in R Studio with R version 4.1.2. Pairwise comparison groups for DEG analysis were assigned as follows: “response to Strep,” “Strep” vs. “mock”; “response to -Strep_2,4-D,” “2,4-D + Strep” vs. “2,4-D”; “response to 2,4-D_Strep,” “2,4-D + Strep” vs. “Strep”; and “response to 2,4-D,” “2,4-D” vs. “mock” (**Figure 1**). Gene ontology (GO) term assignments for annotated genes for DM v6.1 were downloaded from http://spuddb.uga.edu. Additional analyses of differentially expressed genes (DEGs) were performed in R Studio with the following packages: pheatmap (DEG FPKM heat map), VennDiagram (DEG Venn diagrams), and clusterProfiler (GO term enrichment). In GO plots, “obsolete” or select, highly redundant GO terms were removed for clarity.

### 2.3 Fatty acid content analysis

RH89 potato plants were grown as previously described, and at 4 wap, foliage from three plants was sprayed until runoff with each of the following treatments: 200 mg L^−1^ of 2,4-D, 400 mg L^−1^ of 2,4-D, or water (“mock”). Each treatment was supplemented with 0.05% Tween 80. At approximately 11 weeks, tubers for each sample (*n* = 9) were harvested for isolation of approximately 20 g of tuber skins/sample. Lipids were extracted and saponified with 0.5 N of methanolic sodium hydroxide, then methylated with 14% boron trifluoride methanol. Fatty acid methyl esters were extracted via heptane and analyzed for fatty acid content (% by sample mass) via gas chromatography-flame ionization detection (GC-FID). Sample extraction and GC-FID were performed by Eurofins Food Chemistry Testing Madison, Inc. (Madison, WI, USA).

### 2.4 Sucrose content analysis

RH89 potatoes were grown in 6-in. greenhouse pots and inoculated with *Streptomyces* as described above but utilizing strains of either *S. acidiscabies* ATCC49003 or *S. stelliscabiei* NY02-3A, separately. *Streptomyces stelliscabiei* is closely related to *S. caniscabiei*, which was used in the transcriptomic experiment, and disease from all three species is dependent on the thaxtomin A phytotoxin. 2,4-D treatments were performed as described above but included an additional 400 mg L^−1^ treatment group. Tubers were harvested 12 weeks after inoculation (8 weeks post-2,4-D treatment). At least two tubers were pooled from separate individual plants for the collection of each of the four biological replicate tuber periderm and tuber medulla samples from each combination of pathogen inoculum and 2,4-D treatment. Tissue samples were ground in water using Agdia (Elkhart, IN) mesh sample extraction bags. Collected samples were then assayed for sugar content using the Megazymes (Sydney, Australia) Sucrose/D-glucose/D-fructose assay kit following the manufacturer’s recommended protocols, except that samples were syringe-filtered through 0.8 µm filters instead of through filter paper.

## 3 Results

### 3.1 2,4-D treatment attenuated potato common scab disease severity in greenhouse conditions

**Figure 2:**
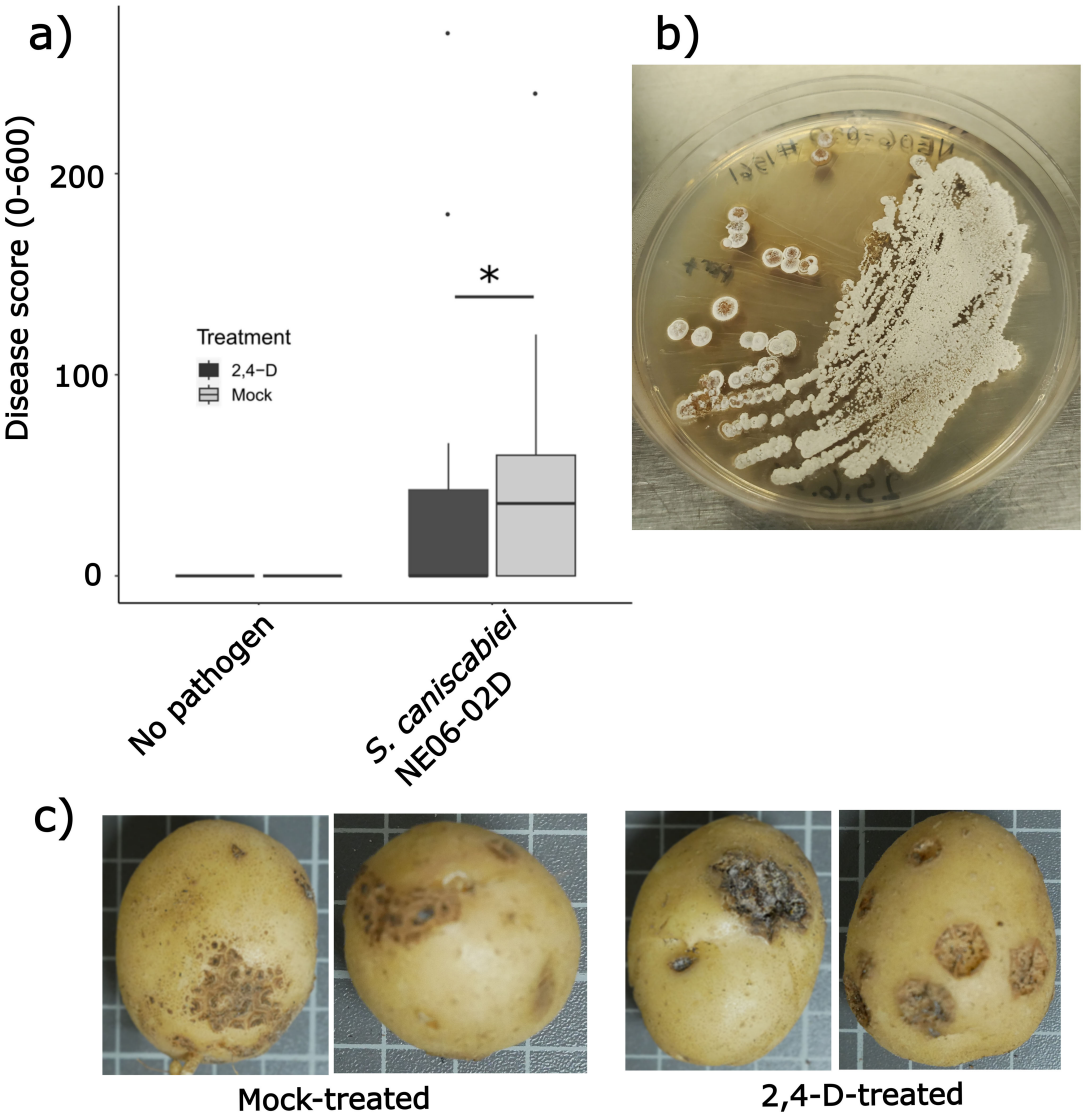
2,4-D treatment significantly attenuates disease symptoms. **(a)** Box plots of disease scores calculated as previously described (Weisberg et al., 2021) comparing non-inoculated plants (left) to pathogen-inoculated plants (right) of both mock-treated (light gray bars) and 2,4-D-treated (dark gray bars) plants. * indicates significant difference (*p* < 0.05) in the Kruskal–Wallis non-parametric test. Similar results were obtained in a second independent experiment. **(b)** Morphology of *S. caniscabiei* strain NE06-02D on ISP-2 agar plates. **(c)** Typical disease symptoms observed on tubers in 2,4-D-treated and mock-treated plants.

We first sought to develop a greenhouse assay in which the efficacy of 2,4-D leaf sprays for suppressing common scab disease severity could be observed. As noted in previous field trials, low-dose 2,4-D treatments can induce minor alterations to plant foliage, including very minor leaf curling or early flowering (data not shown), and common scab infections of tubers do not present visible phenotypes in leaf tissue. Upon tuber harvesting, tuber samples are surveyed for common scab disease severity, with severe disease characterized by raised, pitted lesions and low-severity disease associated with superficial lesions. Leaf spray treatment with 200 mg L^−1^ of 2,4-D of 4-week-old potato plants growing in pots inoculated with pathogenic *Streptomyces* substantially suppressed common scab severity on tubers harvested 8 weeks following the spray treatment (**Figure 2A**), with tubers from 2,4-D-treated plants exhibiting significantly less severe common scab symptoms (**Figure 2B**). For downstream transcriptomics analyses, to avoid sampling tubers for which *Streptomyces* infection may not have been well established in soil, tubers with reduced disease symptoms, and not asymptomatic tubers, were selected for 2,4-D-treated, *Streptomyces*-inoculated plants. Accordingly, leaf samples for 2,4-D-treated, *Streptomyces*-inoculated plants were selected on the basis of the presence of low-severity disease symptoms observed in their corresponding tubers.

### 3.2 RNA-seq mapping statistics and differential gene expression

To begin to survey the transcriptome in response to 2,4-D, *Streptomyces* (“Strep”), and combinations therein, tuber and leaf samples, each corresponding to the four treatment groups (**Figure 1**), were processed for RNA extraction, total RNA library preparation, and double-stranded sequencing via the Illumina platform. Following quality control trimming of Illumina adapter sequences, the resulting FASTQ files contained an average of 69,419,353 individual reads per sample (**Supplementary Table S1**). For alignment, HISAT2 (Kim et al., 2019) was utilized to map the reads to the doubled monoploid reference genome DM v6.1, with a mean mapping rate, including both primary and secondary mappings, of 82.46% (**Supplementary Table S1**). Prior to determining sets of DEGs, we separately evaluated alignment and pseudoalignment methods to assign read pairs to the reference genome. For alignment, featureCounts (Liao et al., 2014) was utilized to align read pairs to the representative gene models for DM v6.1, resulting in a mean of 25,428,076 read pairs per sample, representing an alignment rate of 72.67%, aligned to features (**Supplementary Table S1**). Comparatively, pseudoalignment via Salmon (Patro et al., 2017) resulted in a pseudoalignment rate of 73.55% (**Supplementary Table S1**). Due to the marginally higher alignment performance of Salmon, we utilized this data set for identifying DEGs. Separately, pseudoaligned read pairs from tuber (*n* = 12) and leaf (*n* = 10) samples were analyzed and normalized for differential expression via DESeq2 (Love et al., 2014) in four pairwise comparisons: “response to Strep,” “response to Strep (2,4-D),” “response to 2,4-D,” and “response to 2,4-D (Strep)” (**Figure 1A**). For tuber samples, PCA conveyed that PC1 and PC2 explained 56% and 30% of total variance, respectively, and that Mock and 2,4-D samples were most closely related; Strep and 2,4-D + Strep samples formed additional, separate clusters, with concordance among biological replicates within each treatment group (**Figure 1B**). Leaf sample PCA resulted in PC1 and PC2 explaining 57% and 19% of total variance, respectively, and illustrated clustering of 2,4-D + Strep and 2,4-D samples separate from the Mock and Strep samples (**Figure 1B**). Generally, large DEG counts were observed for all treatment group comparisons except for tuber “response 2,4-D” and leaf “response to Strep (2,4-D)” (**Supplementary Figure S1**).

### 3.3 Transcriptional response to *Streptomyces scabiei* in tuber and leaf tissue

**Figure 3:**
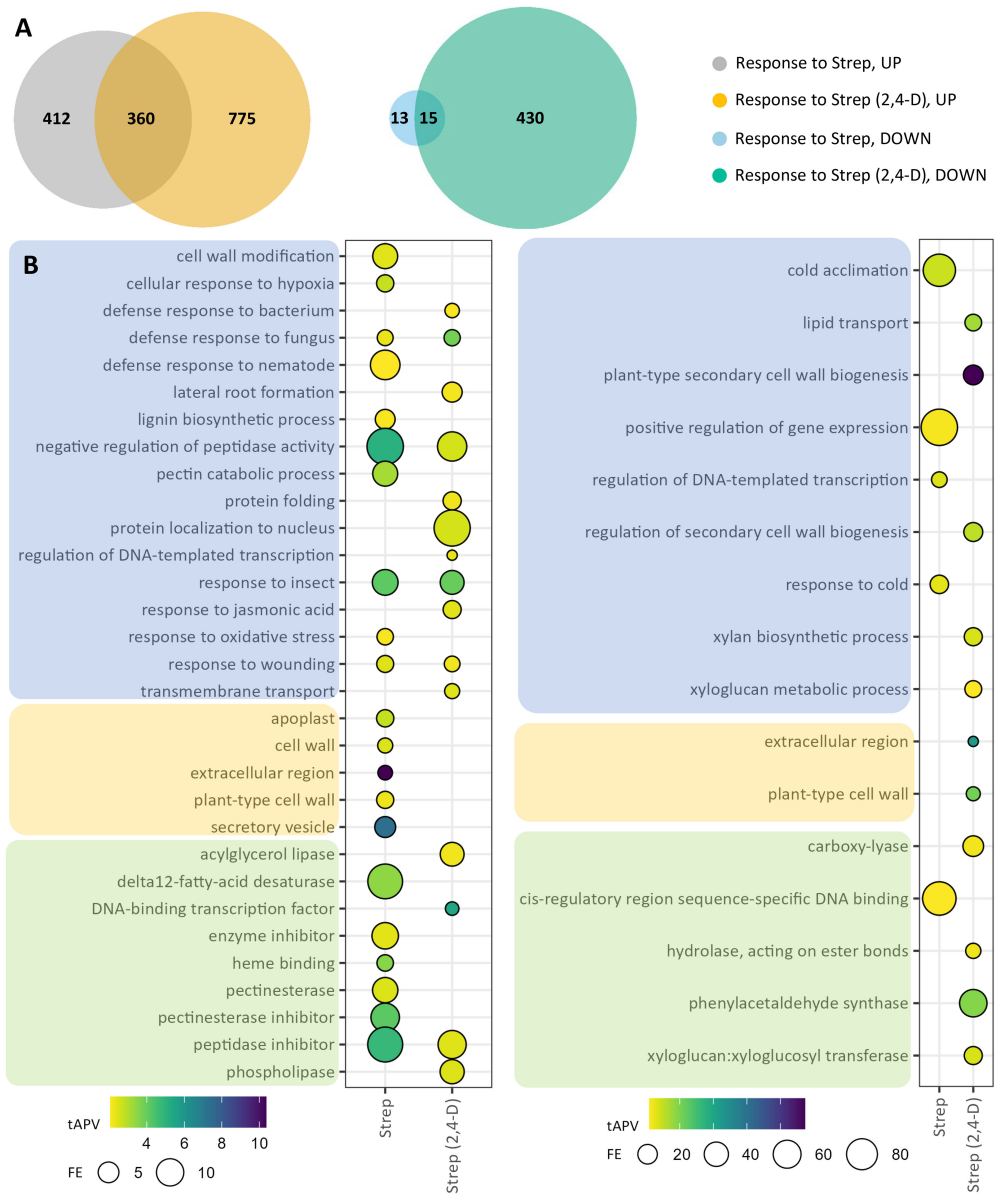
Differentially expressed genes (DEGs) and gene ontology (GO) enrichments for tuber samples in the response to Strep. DEGs were selected on the basis of |log2(fold change)| >2 and adjusted *p*-value <0.05 for the two comparison groups: “response to 2,4-D” and “response to 2,4-D (Strep).” **(A)** Venn diagrams for upregulated (“UP,” left) and downregulated (“DOWN,” right) DEGs. **(B)** GO enrichments for upregulated (left) and downregulated (right) DEGs. Enriched GO terms are grouped into “biological process” (blue), “cellular component” (orange), and “molecular function” (green) domains.

In the tuber sample “response to Strep” comparison, we identified 800 DEGs, consisting of 772 upregulated genes and 28 downregulated genes (**Figure 3A**; **Supplementary Table S2**). Furthermore, response to Strep was also assessed via a comparison of the two groups of 2,4-D treated samples [“response to Strep (2,4-D),” i.e., response to *Streptomyces* infection in the context of 2,4-D treatment], yielding 1,580 DEGs, consisting of 1,135 and 445 upregulated and downregulated genes, respectively (**Figure 3A**; **Supplementary Table S3**). Overlap analysis of the DEGs from these two comparisons illustrated a specific response to *Streptomyces*, including 775 upregulated genes and 430 downregulated genes, in the context of foliar 2,4-D treatment, indicative of a possible enhancement to transcriptional sensitivity in tubers by foliar treatment with 2,4-D. To analyze the transcriptomic response to *Streptomyces* infection in tubers, we incorporated GO assignments for the DEGs identified in “response to Strep” and “response to Strep (2,4-D),” then performed GO enrichment analysis. Among upregulated DEGs in “response to Strep” were significantly enriched GO terms associated with pathogen defense, such as “response to wounding,” “cell wall modification,” “defense response to fungus,” “response to oxidative stress,” and the cellular component term “extracellular region,” with underlying DEGs corresponding to products such as pathogenesis-related (PR) proteins, chitinases, peroxidases, and jasmonate (JA) and ethylene (ET) pathway proteins (**Figure 3B**; **Supplementary Tables S2**, **S4**). Furthermore, several enriched GO terms were associated with fatty acid biosynthesis, with underlying genes corresponding to lipases and fatty acid desaturases. Downregulated DEGs contributed to five enriched GO terms, largely corresponding to underlying dehydration response element-binding (DREB) proteins. Analysis of upregulated DEGs in “response to Strep (2,4-D)” identified enriched GO terms largely overlapping with those from “response to Strep,” with additional terms including “defense response to bacterium,” “response to jasmonic acid,” “phospholipase,” and “acylglycerol lipase” (**Figure 3B**; **Supplementary Table S4**). Furthermore, downregulated DEGs in “response to Strep (2,4-D)” conferred significant enrichment for 13 GO terms, such as “lipid transport,” “extracellular region,” and “cell wall biogenesis.”

Analysis of the systemic response to *Streptomyces* in leaf samples yielded a smaller transcriptional response, as leaf tissue samples were collected early in the tuber development process, prior to the establishment of common scab symptoms. The foliar “response to Strep” comparison yielded 148 upregulated genes and 17 downregulated genes, and 11 upregulated DEGs and 15 downregulated DEGs were identified in “response to Strep (2,4-D)” (**Supplementary Figure S2A**; **Supplementary Tables S5**, **S6**). GO enrichment analysis indicated significant enrichments only from upregulated DEGs in “response to Strep,” with terms corresponding to the apoplast, extracellular matrix, and cell wall, and multiple molecular function terms associated with underlying DEGs encoding germin proteins (**Supplementary Figure S2B**; **Supplementary Tables S5–S7**).

### 3.4 Early, direct transcriptional responses in leaf tissue to foliar 2,4-D

**Figure 4:**
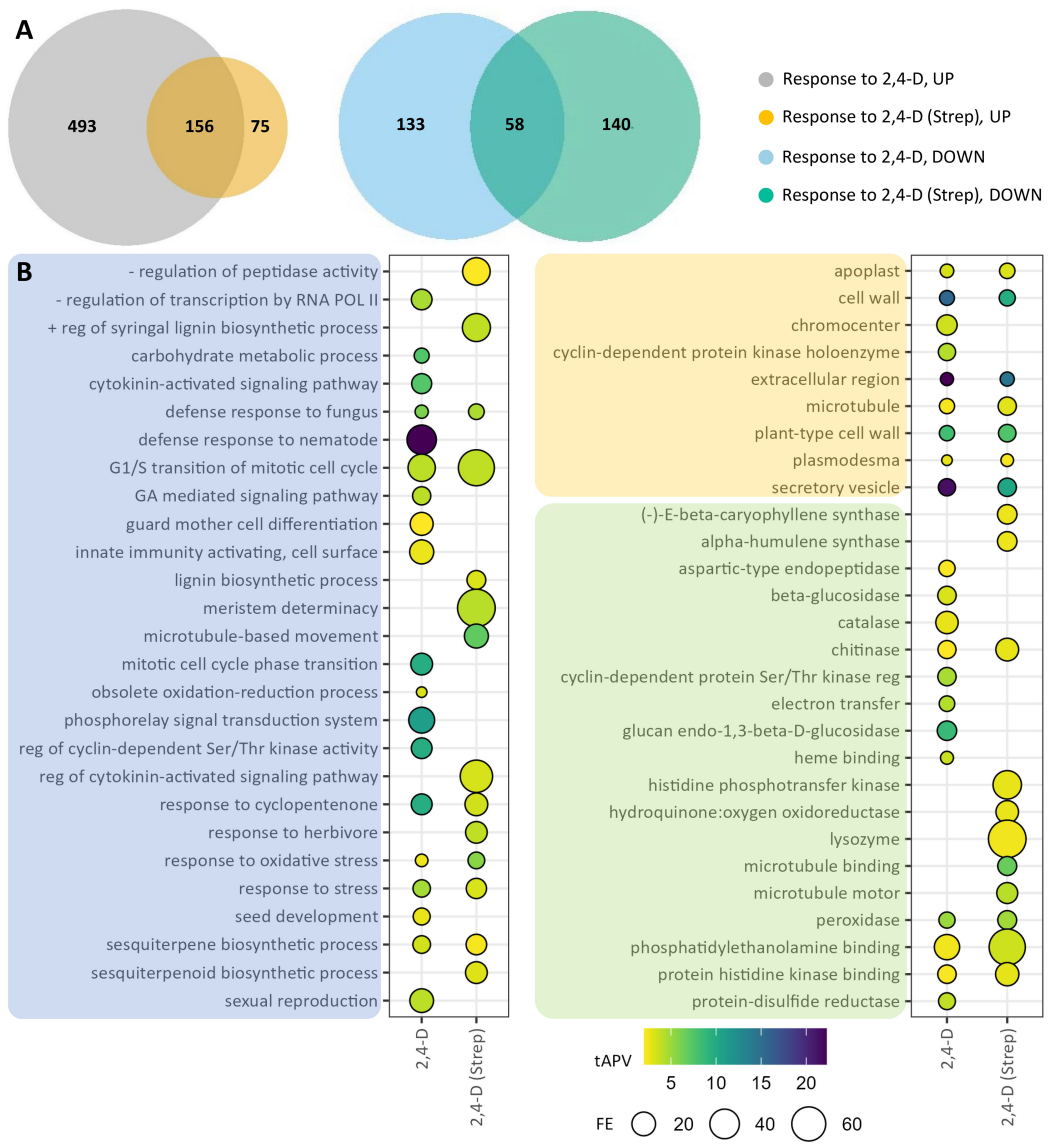
Differentially expressed genes (DEGs) and gene ontology (GO) enrichments for leaf samples in the response to 2,4-D. DEGs were selected on the basis of |log2(fold change) >2 and adjusted *p*-value <0.05 for the two comparison groups: “response to 2,4-D” and “response to 2,4-D (Strep).” **(A)** Venn diagrams for upregulated (left) and downregulated (right) DEGs. **(B)** GO enrichments for upregulated DEGs. Enriched GO terms are grouped into “biological process” (blue), “cellular component” (orange), and “molecular function” (green) domains.

To assess direct transcriptional responses to 2,4-D in leaf tissue, we sampled leaf tissue for RNA-seq 2 days after 2,4-D treatment, corresponding to 8 weeks prior to tuber harvest and preceding the development of common scab symptoms in tuber tissue. Leaf tissue was sampled within the context of *Streptomyces* pathogen in the soil (“response to 2,4-D (Strep)”) and without *Streptomyces* present in the soil (“response to 2,4-D”). In the leaf response to foliar 2,4-D alone, we identified 840 DEGs, consisting of 649 upregulated genes and 191 downregulated genes (**Figure 4A**; **Supplementary Table S8**). Of the 231 upregulated DEGs in “response to 2,4-D (Strep),” 156 overlapped with “response to 2,4-D,” as did 58 of 198 downregulated DEGs. Via GO enrichment analysis, pathways activated by 2,4-D corresponded to functions such as growth and differentiation (e.g., “cytokinin-activated signaling pathway,” “G1/S transition of mitotic cell cycle,” “meristem determinacy,” “microtubule binding”), biotic stress response (e.g., “defense response to fungus,” “innate immunity activating, cell surface,” “response to herbivore”), and carbohydrate metabolism (“carbohydrate metabolic process”) (**Figure 4B**; **Supplementary Tables S8–S10**). Accordingly, underlying genes were associated with a variety of protein classes, such as expansins, auxin efflux carriers and response proteins, cyclins, PR proteins, chitinases, peroxidases, and thioredoxins. GO enrichments derived from downregulated genes were limited to seven total terms and included “carbohydrate transport,” associated with underlying SWEET protein genes, as well as “solute:sodium symporter activity” and “positive regulation of programmed cell death” (**Supplementary Figure S3**; **Supplementary Tables S8–S10**).

### 3.5 Foliar 2,4-D induces transcriptional changes in tubers

**Figure 5:**
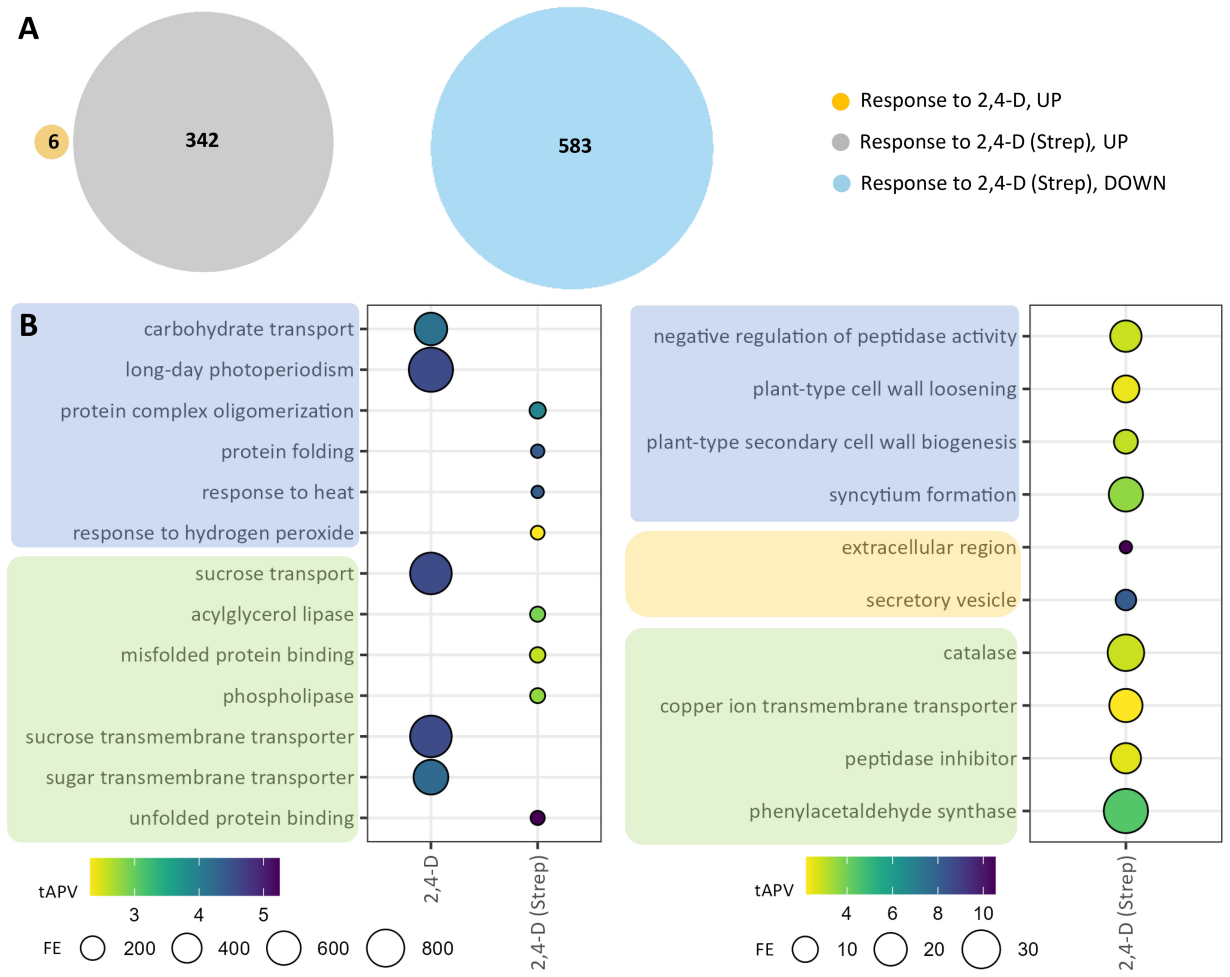
Differentially expressed genes (DEGs) and gene ontology (GO) enrichments for tuber samples in the response to 2,4-D. DEGs were selected on the basis of |log2(fold change)| >2 and adjusted *p*-value <0.05 for the two comparison groups: “response to 2,4-D” and “response to 2,4-D (Strep).” **(A)** Venn diagrams for upregulated (“UP,” left) and downregulated (“DOWN,” right) DEGs. **(B)** GO enrichments for upregulated (left) and downregulated (right) DEGs. Enriched GO terms are grouped into “biological process” (blue), “cellular component” (orange), and “molecular function” (green) domains.

Because foliar 2,4-D treatment enhances resistance to common scab in tubers, we next sought to explore the transcriptional response to foliar 2,4-D treatment, alone and in the context of *Streptomyces* infection, in tuber tissue. 2,4-D was applied early in the tuber development process, and transcriptional response to foliar 2,4-D alone (“response to 2,4-D”) was limited, with only six upregulated DEGs identified (**Figure 5A**; **Supplementary Table S11**). Among these DEGs were two genes corresponding to Nodulin MtN3 family proteins, members of the SWEET family of sucrose transporters, and GO enrichment analysis conveyed strong (363–892-fold) enrichments for five corresponding GO terms, such as “sucrose transport,” from these two genes alone (**Figure 5B**; **Supplementary Tables S11**, **S13**). However, a stronger transcriptional response was observed in the “response to 2,4-D (Strep)” comparison, including 342 upregulated and 583 downregulated DEGs (**Figure 5A**; **Supplementary Table S12**). Eight significantly enriched GO terms were identified from upregulated genes, including “response to hydrogen peroxide,” associated with underlying heat shock protein and WRKY transcription factor genes, as well as “acylglycerol lipase” and “phospholipase” terms corresponding to patatin and phospholipase genes (**Figure 5B**; **Supplementary Tables S12**, **S13**). Downregulated genes contributed to 21 enriched GO terms, including several terms associated with growth, such as “synctium formation” and “plant-type secondary cell wall biogenesis.”

### 3.6 Profiling predicted phenotypic changes in 2,4-D-treated tubers

Among the enriched GO terms identified in the DEGs from tuber responses to 2,4-D were the molecular function terms “acylglycerol lipase” and “phospholipase” (**Figure 5B**; **Supplementary Table S13**), and enrichment for these terms was also observed in 2,4-D-treated tubers in response to *Streptomyces* infection (“response to Strep (2,4-D)”; **Figure 3B**; **Supplementary Table S4**). Accordingly, the DEG sets from these comparison groups were found to include a number of genes with putative lipase, fatty acid desaturase, fatty acid hydroxylase, lipid transfer, and other functions of interest (**Supplementary Tables S3**, **S12**). To probe a potential role for tuber fatty acid metabolic profiles in the systemic response to foliar 2,4-D, we analyzed fatty acid contents in tuber tissue harvested from plants treated with 200 mg L^−1^ of 2,4-D, 400 mg L^−1^ of 2,4-D, or mock treatment (**Supplementary Table S14**). Esterified fatty acids from tuber skin samples were quantified via the GC-FID panel; however, most classes of fatty acids were below the detection limit, potentially indicating insufficient sensitivity of lipidomic profiling, and no significant differences between treatment groups were identified.

Upregulated DEGs in the tuber response to 2,4-D contributed to GO term enrichments associated with sucrose transport (**Figure 5B**; **Supplementary Table S13**), and among upregulated tuber DEGs in “response to Strep (2,4-D),” “response to 2,4-D,” and “response to 2,4-D (Strep)” were genes corresponding to SWEET proteins and sucrose synthases (**Supplementary Tables S3**, **S11**, **S12**). Therefore, we analyzed tubers for sucrose, glucose, and fructose content separately in both periderm and medulla tissue following spray treatment with 200 mg L^−1^ of 2,4-D, 400 mg L^−1^ of 2,4-D, or mock treatment. Tuber samples were collected at harvest, 12 weeks after inoculation of the potting mix with a mock inoculum, *S. stelliscabiei* strain NY02-3A, or *S. acidiscabies* strain ATCC49003 (**Supplementary Figure S4**). While 2,4-D treatments did lead to increased abundance of sucrose in both periderm and medulla tissue, sucrose was detected only at low levels in the periderm (the site of infection), and there was no significant dosage effect for any inoculation condition–tissue type combination based on the Kruskal–Wallis non-parametric rank-sum test. Treatment with 2,4-D significantly increased glucose abundance in the medulla and fructose abundance in the medulla and periderm tissue of mock-inoculated plants. However, these increases in glucose and fructose were not observed in pathogen-inoculated tubers.

## 4 Discussion

Multifactor transcriptional profiling of potato leaf and tuber tissue response to 2,4-D treatment and pathogen infection revealed multiple candidate pathways for elucidating the mechanisms through which low-dose 2,4-D treatments attenuate common scab disease severity. While previous research has focused on elucidating the impact of 2,4-D on tuber physiology, pathogen inhibition, and thaxtomin A uptake and toxicity (Tegg et al., 2008, 2012, 2013, 2016), this study sought to identify potato global transcriptomic responses to 2,4-D in the context of pathogen infection. Foliar 2,4-D treatments substantially altered transcriptional responses to the pathogen, and likewise, *Streptomyces* infection substantially altered transcriptional responses to 2,4-D treatments. In general, stronger transcriptional responses were seen in plants both treated with 2,4-D and infected with pathogen than either variable in isolation. Additionally, the transcriptomic response to 2,4-D treatment was only partially overlapping between the pathogen-inoculated and mock-inoculated samples, demonstrating the need for considering both experimental factors in parallel to more fully understand the complex interaction.

We observed that common scab infection caused by *S. caniscabiei*, which causes more raised lesions and fewer pitted lesions compared to the eponymous pathogenic species *S. scabiei* (Weisberg et al., 2021), induced significant transcriptional reprogramming in tuber periderm tissue, and in pathways known to be involved in pathogen defense, as previously observed following infection by the related pathogenic species *S. scabiei* (Fofana et al., 2020; Li et al., 2024) (**Figure 3**; **Supplementary Tables S2–S4**). In the plant response to pathogen alone (e.g., no 2,4-D treatment), the population of upregulated DEGs was enriched for GO terms corresponding to JA signaling and responses to insects, fungus, wounding, and nematodes, affirming transcriptional defense responses to pathogen infection. Such GO terms were also enriched in the plant response to the pathogen in the context of 2,4-D treatments, demonstrating the involvement of these specific, robust transcriptional responses even in tuber periderm tissue with lower disease severity resulting from the disease-suppressing effects of 2,4-D treatment.

Transcriptional response to the pathogen without 2,4-D treatment included numerous overlapping GO terms and DEGs with the transcriptional response to the pathogen with 2,4-D treatment. However, there were also many unique DEGs and GO terms in each separate analysis and a larger number of enriched GO terms and significant DEGs in response to the pathogen when the plants were previously treated with 2,4-D. This finding suggests that 2,4-D treatment elicits durable effects on the transcriptome, including cumulative effects from lower disease pressure, despite the only difference being a single 2,4-D treatment 8 weeks prior to harvest. This single treatment regimen is the current agricultural practice for low-dose foliar 2,4-D sprays to induce anthocyanin production in red potato production and is also associated with reduced levels of common scab. Examining enriched GO terms and differentially regulated DEGs that appear in the “response to Strep” comparison group but not when tubers that had been treated with 2,4-D (“response to Strep (2,4-D)”) may reveal transcriptional responses attenuated by 2,4-D, as evidenced by GO terms related to multiple classes of enzymatic inhibitors. Interestingly, several additional GO terms related to fatty acid metabolism were enriched in the tuber response to the pathogen only in the context of 2,4-D treatment, suggesting a role for fatty acids in 2,4-D-induced disease resistance. Fatty acids have been demonstrated to have critical roles in plant defenses against other pathogens, through fatty acid signaling pathways, for example (Kachroo and Kachroo, 2009; Pretorius et al., 2021). However, no differences in fatty acid content were measured in tuber periderm samples from 2,4-D-treated and mock-treated potatoes (**Supplementary Table S14**). Tuber periderm samples for fatty acid analysis were collected 7 weeks after 2,4-D treatment, at the time of tuber harvest and disease scoring. It is possible that fatty acid profiles and signaling activity may have been altered only in younger tubers, a developmental stage that serves as the critical window for common scab infection (Khatri et al., 2011), in the 2,4-D-treated plants.

Unsurprisingly, pathogen infection led to a comparatively smaller transcriptional response in leaf tissue in the “response to Strep” comparison group, as leaf tissue was responding systemically and before the establishment of common scab disease systems (**Supplementary Figure S2**; **Supplementary Tables S5–S7**). Multiple genes encoding Germin-like proteins, which have been linked with JA signaling and pathogen defense (Pei et al., 2019; To et al., 2022), were upregulated in foliar response to *Streptomyces* infection. However, 2,4-D treatment induced robust transcriptional changes in leaf tissue that were partially overlapping between the experiments with (“response to 2,4-D (Strep)”) and without (“response to 2,4-D”) pathogen infection, and the overall transcriptional response was largely represented by upregulated genes (**Figures 4**, **Supplementary Figure S3**; **Supplementary Tables S8–S10**). Analysis of the transcriptional responses validated the auxin and cytokinin analog functionality of 2,4-D by conferring multiple enriched GO terms related to cell growth, development, and differentiation; meristem determinacy; cytokinin signaling; and microtubule function. Upregulated DEGs and associated enriched GO terms also suggest potential immune priming by 2,4-D in leaf tissue; enhanced expression of genes corresponding to PR proteins, chitinases, thioredoxins, and peroxidases, for example, was found to be upregulated in the leaves following 2,4-D treatment. These results suggest that 2,4-D may function as an effective foliar immune priming agent, as demonstrated for multiple low-dose herbicide treatments (Martinez et al., 2018). Future work incorporating a more granular time course evaluation of potato response to 2,4-D could help to further establish the potential efficacy of 2,4-D as an immune priming agent and determine how alterations to the foliar transcriptome influence tuber development and defense against soil diseases such as common scab.

In tuber samples, while 2,4-D was shown to influence transcriptional reprogramming in response to *Streptomyces* infection (“response to Strep (2,4-D)”), the response to 2,4-D, with (“response to 2,4-D (Strep)”) and without (“response to 2,4-D”) the background of *Streptomyces* infection, elicited a less robust transcriptional response (**Figure 5**; **Supplementary Tables S11–S13**). Although 2,4-D treatments were applied 8 weeks prior to the collection of tuber periderm samples for RNA analysis, thereby possibly limiting the observable transcriptional response upon tuber harvesting, 2,4-D is thought to translocate and accumulate in tubers (Burrell, 1982; Tegg et al., 2008). The most notable transcriptional change in tuber periderm tissue induced by 2,4-D without the presence of pathogen infection was the presence of enriched GO terms associated with significant upregulation of Nodulin MtN3/SWEET genes, a gene class encoding proteins involved in the transport of sucrose and other carbohydrates and also shown to play critical roles in pathogen defense (Denancé et al., 2014; Breia et al., 2021). Interestingly, SWEET genes were largely downregulated in leaf tissue following 2,4-D treatment, conveying the possibility that 2,4-D influences the source–sink relationship for sugars in leaf and tuber tissue, respectively (Ludewig and Flügge, 2013). Some of the corresponding SWEET genes, as well as several sucrose synthases and invertases, were also found to be upregulated in the tuber response to *Streptomyces* infection (**Supplementary Tables S2** and **S3**), suggesting a role for sugar transport and metabolism in the 2,4-D-induced enhancement to common scab resistance. Sucrose has been implicated in defense response and immune priming in other pathosystems and is proposed to be a mobile defense signal, elicitor of defense response, and a critical component of carbohydrate availability, which impacts pathogen growth at the plant–pathogen interface (Gómez-Ariza et al., 2007; Morkunas and Ratajczak, 2014; Li et al., 2017; Jeandet et al., 2022; Liu et al., 2022). Foliar applications of 2,4-D did lead to a moderate, but not significant, increase in sucrose content in both tuber periderm and medulla tissue for both mock-inoculated and pathogen-inoculated samples (**Supplementary Figure S4**). The reducing sugar hexamers glucose and fructose accumulated at significantly higher levels in tuber periderm and medulla tissue from 400 mg L^−1^ 2,4-D-treated, mock-inoculated plants; however, this trend was not observed in tubers from pathogen-inoculated plants. Pathogen infection did not significantly increase sugar levels in the Strep-inoculated plants, in contrast to a previous work, suggesting an increase in tuber periderm reducing sugars due to common scab infection (Goto, 1981). Similar to caveats for the interpretation of the analysis of fatty acid content in tubers following 2,4-D treatment, sucrose and other carbohydrate content or signaling may be more significantly altered due to 2,4-D treatment in young tubers during the critical window of scab infection. More rigorous time-course sampling may reveal notable 2,4-D-induced phenotypic alterations, such as sugar abundance, fatty acid content, and periderm structure, in young tubers during initial *Streptomyces* infection.

When 2,4-D treatments were applied in the background of *Streptomyces* infection, substantially more DEGs and enriched GO terms were identified from tuber samples (**Figure 5**). Among upregulated DEGs were several genes encoding patatins, which have been implicated in disease resistance in potato and other *Solanaceae* vegetable crops potentially through impacts on storage and lipid metabolism (Kim et al., 2014; Bártová et al., 2019; Cheng et al., 2019). Patatins were also found to accumulate to much higher levels in a potato somaclone habituated with thaxtomin A for improved resistance to common scab (Isayenka et al., 2023). Additional phospholipase genes were also significantly upregulated, further suggesting a reprogramming of fatty acid metabolism following 2,4-D treatment. Surprisingly, the most downregulated DEG across the entire experimental platform, identified as highly downregulated in both the tuber “resp to Strep (2,4-D)” (**Supplementary Table S3**) and “resp to 2,4-D (Strep)” (**Supplementary Table S12**) analyses, was the TIR-NBS-LRR class putative *R* gene Soltu.DM.S001640. Why the combination of 2,4-D foliar application with *Streptomyces* infection led to the extreme downregulation of this putative *R* gene remains to be elucidated.

## 5 Conclusions

Multifactor analysis of potato leaf and tuber response to 2,4-D foliar application and *Streptomyces* infection revealed significant transcriptional reprogramming associated with reduced disease severity in 2,4-D-treated plants. Transcriptional analysis of both pathogen infection and chemical treatment facilitated the identification of differentially expressed genes and gene classes associated not only with host responses to the pathogen or 2,4-D treatment alone but also with the responses to the combined 2,4-D treatment and *Streptomyces* infection. Numerous gene pathways associated with canonical plant immunity and wounding response were upregulated in response to *Streptomyces*, affirming that multiple canonical defense pathways are being activated in response to common scab infection. Treatment with 2,4-D upregulated many genes in overlapping, canonical defense pathways, as well as in other pathways associated with immune priming. Additional phenotypic characterization of 2,4-D-treated potato is necessary to validate the molecular mechanisms responsible for 2,4-D-induced common scab disease resistance. Because 2,4-D and other auxin analog treatments also have efficacy against other potato pathogens, similar characterization of the molecular impacts of such auxin analog treatments in other potato pathosystems may reveal broad-spectrum disease resistance mechanisms.

## Data Availability

The datasets presented in this study can be found in online repositories. The names of the repository/repositories and accession number(s) can be found in the article/**Supplementary Material**.
