# Supplementary material for: Multifactor transcriptional profiling of potato during 2,4-D-induced resistance to common scab disease
Source: Front Plant Sci. 2025 Aug 18;16:1641317. doi: 10.3389/fpls.2025.1641317 (PMC12399720; doi:10.3389/fpls.2025.1641317)
Supplement: Supplementary file 1 [file DataSheet1.pdf]

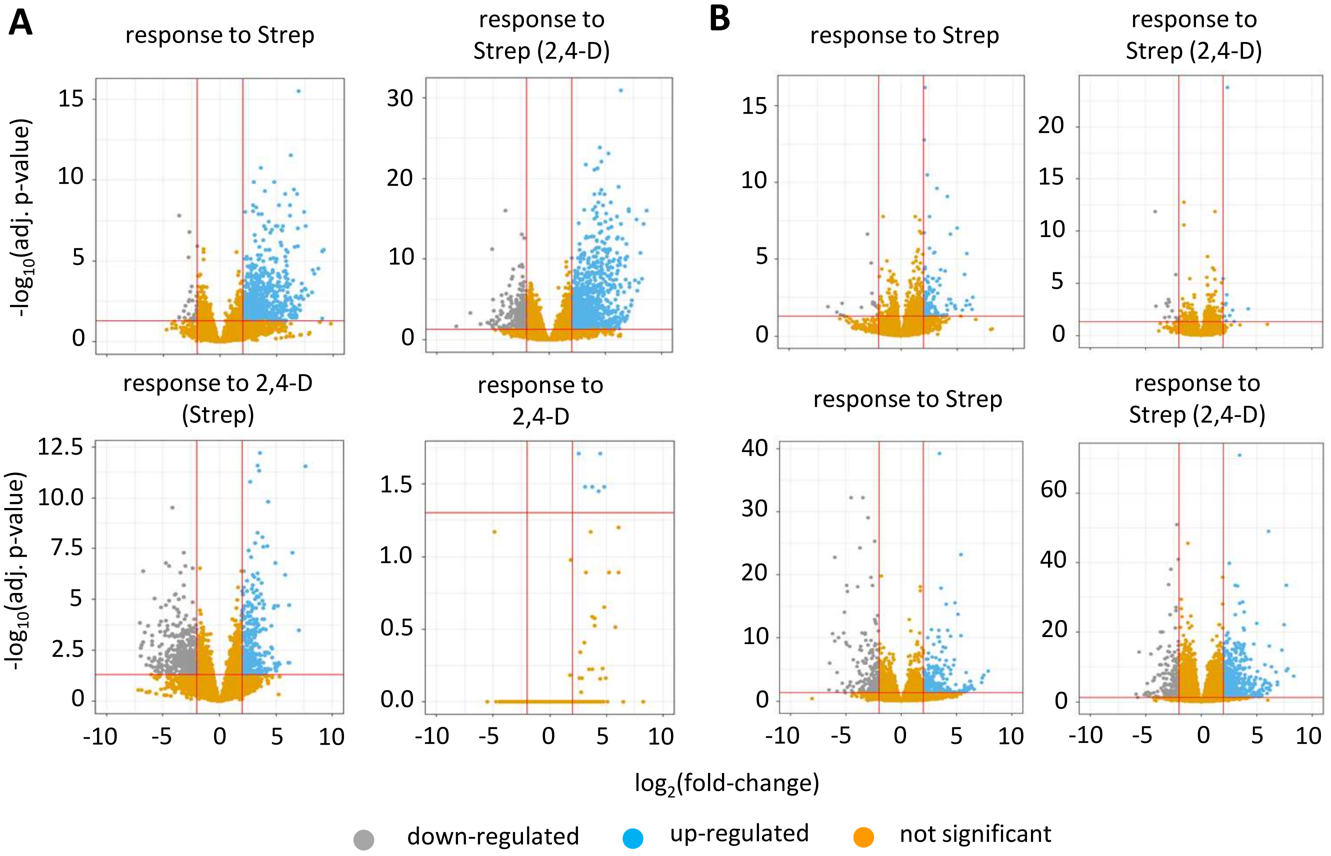

Figure S1. Volcano plots for differentially expressed genes (DEGs) from tuber (A) and leaf (B). Red lines indicate an adjusted p-value threshold of 0.05 (horizontal) and $\log_2$(fold-change) thresholds of |2| (vertical).

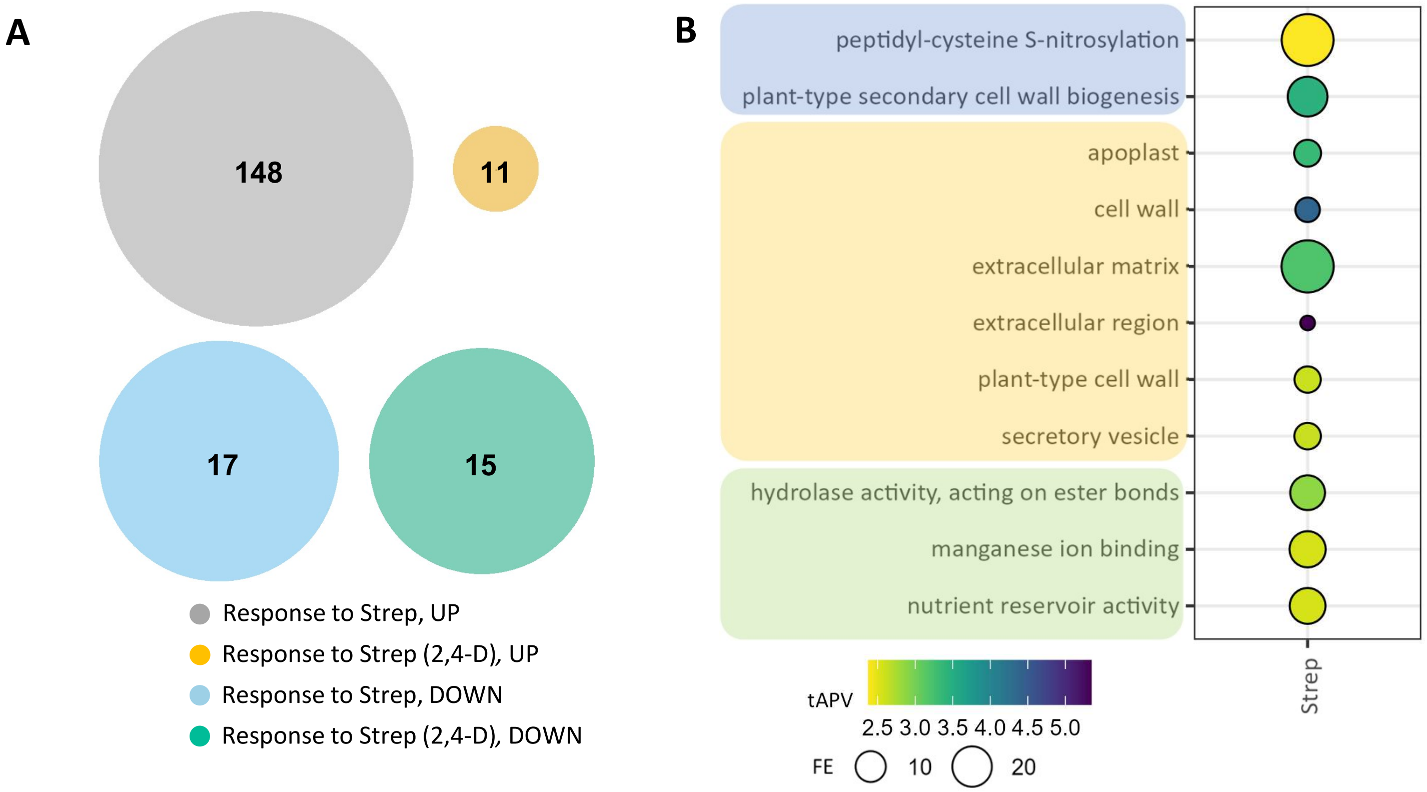

Figure S2. Differentially Expressed Genes (DEGs) and Gene Ontology (GO) enrichments for leaf samples in the response to Strep. DEGs were selected on the basis of |log2(fold-change)| > 2 and adjusted p-value < 0.05 for the two comparison groups, “response to Strep” and “response to Strep (2,4-D)”. (A), Venn diagrams for upregulated (“UP”, upper) and downregulated (“DOWN”, lower) DEGs. (B), GO enrichments for upregulated DEGs. Enriched GO terms are grouped into “biological process” (blue), “cellular component” (orange), and “molecular function” (green) domains.

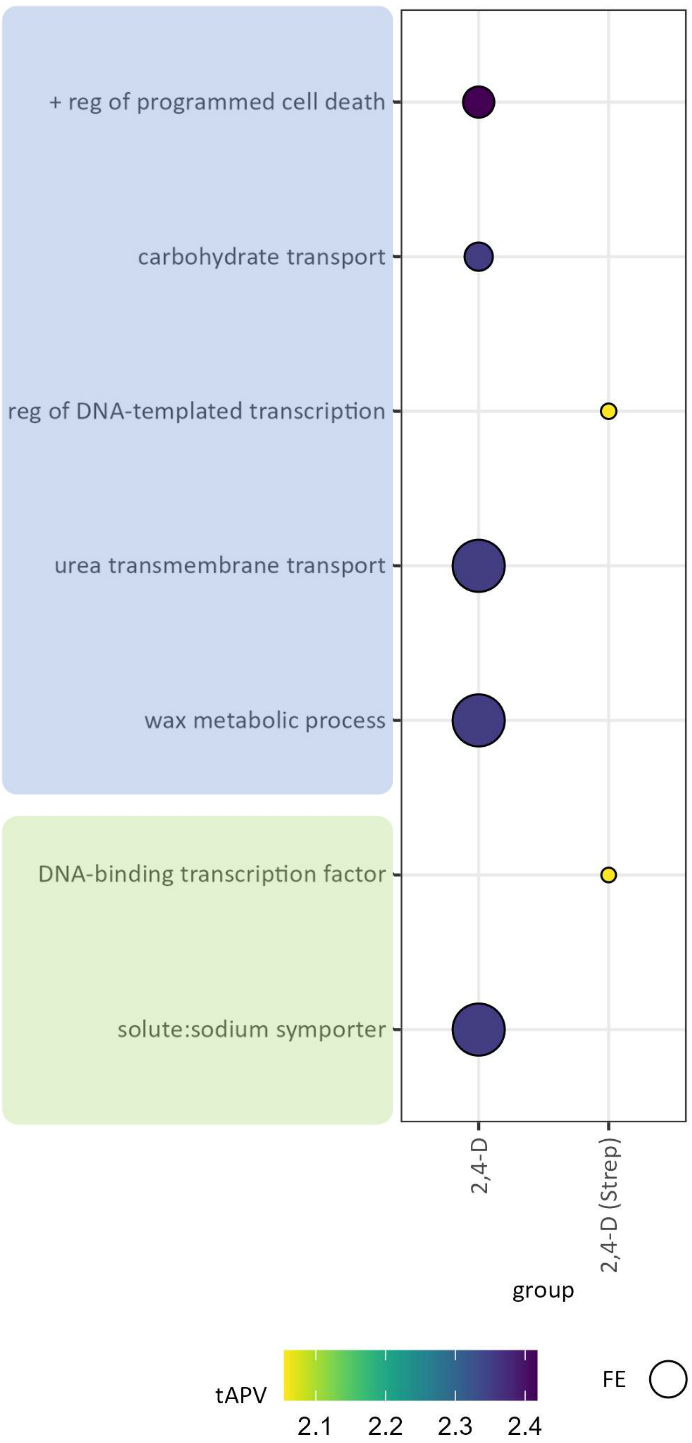

Figure S3. Gene Ontology (GO) enrichments for downregulated differentially expressed genes (DEGs) in the leaf response to 2,4-D. DEGs were selected on the basis of |log2(fold-change)| > 2 and adjusted p-value < 0.05 for the two comparison groups, "response to 2,4-D" and "response to 2,4-D (Strep)". Enriched GO terms are grouped into "biological process" (blue) and "molecular function" (green) domains.

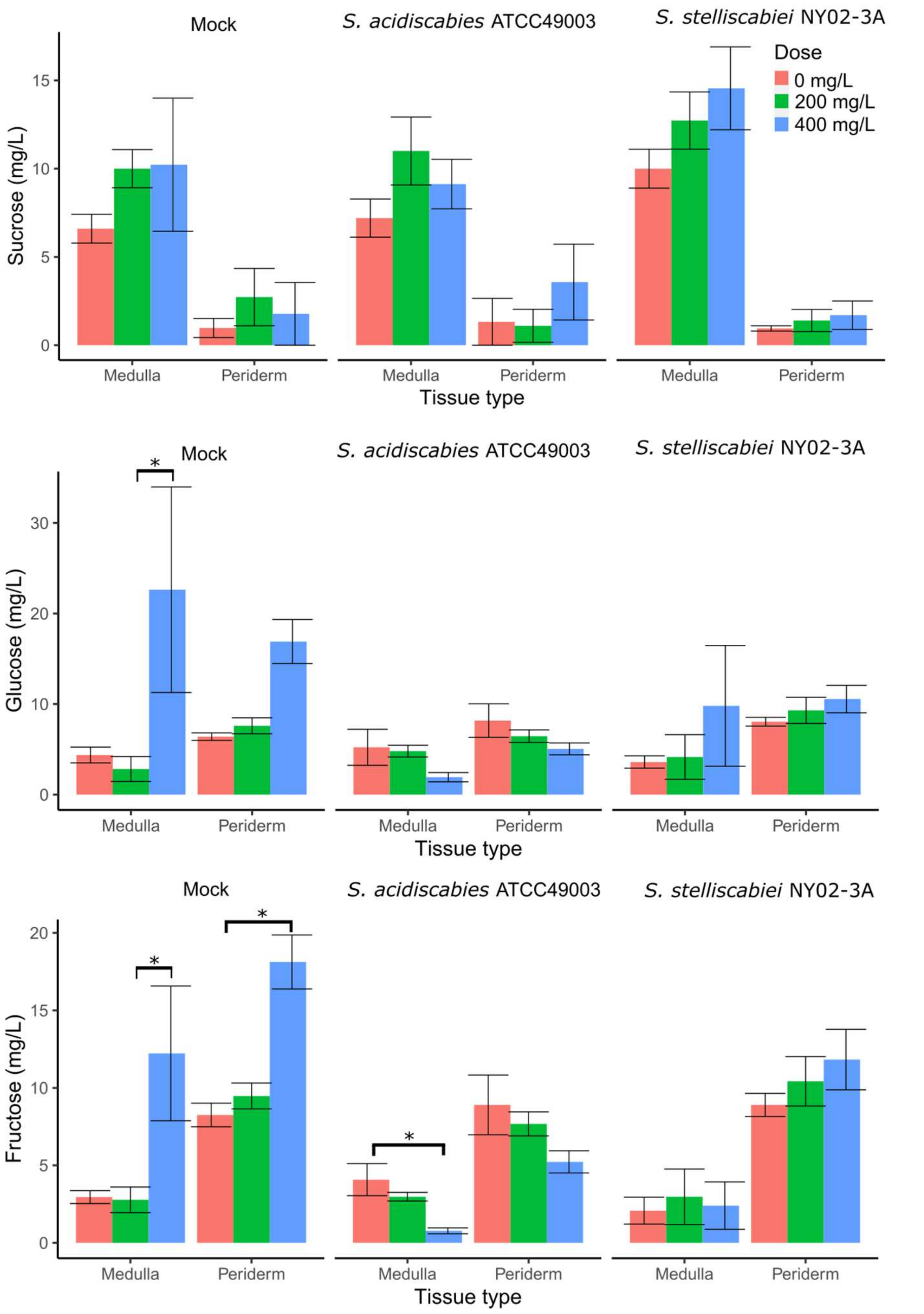

Figure S4. Abundance of sucrose, glucose, and fructose in tuber periderm and medulla tissue following treatment with either 0 mg/L 2,4-D (red bars), 200 mg/L 2,4-D (green bars), or 400 mg/L 2,4-D (blue bars). Samples were taken at harvest, 12 weeks after inoculation with either mock vermiculite or indicated bacterial strains. Asterisks indicate significance in a pairwise Dunn's test ($\alpha=0.05$) with Bonferroni correction. Only tissue/strain datasets for which Kruskal-Wallis tests indicated significant dosage effects ($p<0.05$) were considered for Dunn's tests.
